# Seroprevalence of *Toxoplasma gondii* infection in water buffaloes (*Bubalus bubalis*) in Veracruz State, Mexico and its association with climatic factors

**DOI:** 10.1186/s12917-014-0232-5

**Published:** 2014-09-30

**Authors:** Cosme Alvarado-Esquivel, Dora Romero-Salas, Zeferino García-Vázquez, Anabel Cruz-Romero, Álvaro Peniche-Cardeña, Nelly Ibarra-Priego, Mariel Aguilar-Domínguez, Adalberto A Pérez-de-León, Jitender P Dubey

**Affiliations:** Biomedical Research Laboratory, Faculty of Medicine and Nutrition, Juárez University of Durango State, Avenida Universidad S/N, 34000 Durango, Dgo Mexico; Laboratorio de Parasitología, Facultad de Medicina Veterinaria y Zootecnia, Universidad Veracruzana, Circunvalación y Yáñez S/N, 91710 Veracruz, Mexico; Centro Nacional de Investigación Disciplinaria en Parasitología Veterinaria, INIFAP, Carretera Federal Cuernavaca-Cuautla No. 8534, C.P. 6225 Jiutepec, Morelos México; United States Department of Agriculture, Agricultural Research Service, Knipling-Bushland U.S. Livestock Insects Research Laboratory, 2700 Fredericksburg Road, Kerrville, TX 78028 USA; United States Department of Agriculture, Agricultural Research Service, Beltsville Agricultural Research Center, Animal Parasitic Diseases Laboratory, Building 1001, Beltsville, MD 20705-2350 USA

**Keywords:** Buffaloes, *Toxoplasma gondii*, Seroprevalence, Epidemiology, Mexico

## Abstract

**Background:**

Infection with *Toxoplasma gondii* in water buffaloes (*Bubalus bubalis*) is of epidemiological importance because of the risk for transmission to humans. We sought to determine the seroprevalence of *T. gondii* infection in 339 water buffaloes in Veracruz State, Mexico using the modified agglutination test (MAT, cut off 1:25). Seroprevalence association with general characteristics of buffaloes and their environment was also investigated.

**Results:**

Antibodies to *T. gondii* were found in 165 (48.7%) of the 339 buffaloes with MAT titers of 1:25 in 104, 1:50 in 52, and 1:100 in 9. Bivariate analysis showed that seroprevalence of *T. gondii* infection was similar in buffaloes regardless of their general characteristics i.e., age, sex, and breed. In contrast, the seroprevalence in buffaloes varied significantly with environmental characteristics including altitude, mean annual temperature, and mean annual rainfall of the municipalities studied. Multivariate analysis showed that *T. gondii* seropositivity in buffaloes was associated with a mean annual rainfall between 1266–1650 mm (OR = 1.84; 95% CI: 1.15-2.94; *P* = 0.01).

**Conclusions:**

Results indicate that environmental characteristics may influence the seroprevalence of *T. gondii* infection in buffaloes. This is the first report on the seroprevalence of *T. gondii* infection in buffaloes in Mexico. Further research is needed to assess the risk for infection in humans associated with the ingestion of raw or undercooked meat from buffaloes infected with *T. gondii*.

## Background

*Toxoplasma gondii* is a widely distributed parasite infecting warm-blooded animals including the water buffalo (*Bubalus bubalis*) [1]. Toxoplasmosis in humans may be a severe disease affecting the lymph nodes, eyes, and central nervous system and can be life-threatening mostly in immunocompromised patients. Water buffaloes are important to the economy of many countries and in Mexico they represent an emerging alternative for diversified livestock production [2]. In Asia, water buffaloes are the major source of milk and milk products, and their meat is consumed by humans in several countries. In general, buffaloes, like cattle, are considered resistant to clinical toxoplasmosis and there is no valid report of isolation of viable *T. gondii* from buffalo meat [1,3]. Recently, Dehkordi et al. [4] reported isolation of viable *T. gondii* from milk of 7 of 164 buffaloes in Iran, but the validity of these results has been questioned [5]. This report has raised concerns regarding transmission of *T. gondii* by consuming raw products from buffaloes.

We are not aware of any report of *T. gondii* infection in water buffaloes from Mexico. The objective of this study was to determine the seroprevalence and correlates of *T. gondii* infection in water buffaloes raised in several municipalities in Veracruz, Mexico.

## Methods

### Water buffaloes surveyed

Water buffaloes (*Bubalus bubalis*) (n = 339) from 2 geographical regions (Olmeca and Papaloapan) of Veracruz, Mexico were sampled from September 2012 to August 2013. These water buffaloes ranged freely and grazed on natural pastures. Sampling was based on accessibility to ranches raising buffaloes in the Mexican state of Veracruz that shares its eastern border with the Gulf of Mexico. Water buffaloes were located in 6 ranches throughout these 4 municipalities: Isla (18°02’N 95°32’W), Juan Rodriguez Clara (18°00’N 95°24’W), Las Choapas (17°55’N 94°06’W), and Sayula de Alemán (17°53’N 94°57’W). A questionnaire was used to obtain the general characteristics of the buffalo herds including age, sex, weight, breed, and obstetric history. Additionally, environmental data were recorded including presence of common water source shared with other animals in the ranch, cohabitation with other animals (cats, dogs, cattle), and climatic conditions. The age of the buffaloes ranged from 0.2 to 11 years old, 32 were males, and 307 females. Three water buffalo breeds were represented: Murrah, Carabao, and Jafarabadi.

### Ethics statement

This project was approved by the Bioethics and Animal Welfare Commission of the Facultad de Medicina Veterinaria y Zootecnia of Universidad Veracruzana. Consent was obtained from the owners of the Buffaloes.

### Serological examination for *T. gondii* antibodies

Serum samples were obtained and stored at −20°C until tested. Testing for *T. gondii* antibodies in the buffaloes’ sera was performed using 2-fold serial dilutions from 1:25 to 1:3200 with the modified agglutination test (MAT) as described by Dubey and Desmonts [6]. A titer of 1:25 was used as cut off for seropositivity in the MAT.

### Statistical analysis

Statistical analysis was performed with the aid of Epi Info version 3.5.4. (Centers for Disease Control and Prevention: http://wwwn.cdc.gov/epiinfo/ and SPSS version 15.0 (SPSS Inc. Chicago, Illinois) software. The Pearson’s chi-squared test was used for comparison of the frequencies among groups. The association between the buffaloes’ characteristics and *T. gondii* seropositivity was assessed by multivariable analysis. The dependent variable was seropositivity to *T. gondii* by MAT for an individual animal. Independent variables included in the multivariable analysis were those with a *P* value ≤0.10 in the bivariate analysis: region, municipality, altitude, mean annual temperature, mean annual rainfall, and common water source. Odds ratio (OR) and 95% confidence interval (CI) were calculated by multivariate analysis using backward stepwise logistic regression analysis. The Hosmer-Lemeshow goodness of fit test was used to assess the fitness of the regression model. Statistical significance was set at a *P* value of < 0.05.

## Results

Antibodies to *T. gondii* were found in 165 (48.7%) of the 339 buffaloes with MAT titers of 1:25 in 104, 1:50 in 52, and 1:100 in 9; none of the samples had a higher titer. Seroprevalence of *T. gondii* infection was significantly (*P* = 0.02) higher in buffaloes in the Olmeca region (53.3%) than in the Papaloapan region (40.8%). All ranches had seropositive buffaloes, and seroprevalence in buffaloes varied significantly (*P* = 0.009) among ranches (36.2%-63.8%).

A correlation of *T. gondii* seropositivity rates and environmental characteristics is shown in Table 1. The seroprevalence varied among municipalities (*P* = 0.002). The seroprevalence of *T. gondii* infection was significantly (*P* = 0.003) higher in buffaloes at 80–95 meters above sea level (121/222, 54.5%) than those at 10–60 meters above sea level (44/117, 37.6%). The seroprevalence in warm climate (142/280, 50.7%) was similar to the one in warm-humid climate (23/59, 39.0%) (*P* = 0.10). In contrast, the seroprevalence was significantly higher in municipalities with 27°C of mean annual temperature (114/214, 53.3%) than in those with 24.9-25°C mean annual temperature (51/125, 40.8%) (*P* = 0.02). In addition, seroprevalence varied among mean annual rainfall, the seroprevalence was significantly (*P* = 0.003) higher in municipalities with 1266–1650 mm of mean annual rainfall (121/222, 54.5%) than those with 2316–2900 mm of mean annual rainfall (44/117, 37.6%).

**Table 1:** **Seroprevalence of** ***T. gondii*** **infection in water buffaloes in Veracruz, Mexico**

		**Meters above sea level (masl)** ^**c**^		**Mean annual temperature** **(matp)** ^**d**^	**Mean annual** **rainfall (mar)** ^**e**^	**Buffaloes tested**	**Seropositivity to** ***T. gondii***	
**Region** ^**a**^	**Municipality** ^**b**^		**Climate**			**No.**	**No.**	**%**
Olmeca	Las Choapas	10	Warm	27.0°C	2900 mm	58	21	36.2
	Sayula de Alemán	80	Warm	27.0°C	1650 mm	156	93	59.6
	All					214	114	53.3
Papaloapan	Isla	60	Warm-humid	24.9°C	2316 mm	59	23	39.0
	J. Rodriguez Clara	95	Warm	25.0°C	1266 mm	66	28	42.4
	All					125	51	40.8
All						339	165	48.7

^a^Statistically significant difference in seroprevalences among regions (*P =* 0.02).

^b^Statistically significant difference in seroprevalences among municipalities (*P =* 0.002).

^c^Statistically significant difference in seroprevalences among masl (*P =* 0.002).

^d^Significantly higher seroprevalence at 27°C (53.3%) than at 24.9-25°C (40.8%) matp (*P =* 0.02).

^e^Statistically significant difference in seroprevalences among mar (*P =* 0.002).

Table 2 shows the correlation of *T. gondii* seropositivity rates and the general characteristics of the buffaloes. Seroprevalence of *T. gondii* infection did not vary with age, sex, breed, weight, and type of buffaloes, and with sharing of water, or contact with cattle. The seroprevalence of *T. gondii* infection was similar in female buffaloes with abortion history than in female buffaloes without abortion history. All buffaloes had contact with cats and dogs.

**Table 2:** **Correlation of general characteristics of the 339 buffaloes and seroprevalence of** ***T. gondii*** **infection**

	**Buffaloes tested**	**Seroprevalence of** ***T. gondii*** **infection**		
**Characteristics**	**No.**	**No.**	**%**	***P*** **value**
Age (years)				
<1	15	9	60	0.45
1-2	119	60	50.4	
3-4	113	52	46	
5-6	53	29	54.7	
7-11	39	15	38.5	
Sex				
Male	32	19	59.4	0.2
Female	307	146	47.6	
Breed				
Murrah	192	95	49.5	0.93
Carabao	91	43	47.3	
Jafarabadi	56	27	48.2	
Weight (kg)				
100-200	17	11	64.7	0.6
201-300	107	52	48.6	
301-400	60	27	45	
401-500	61	32	52.5	
>501	94	43	45.7	
Animal type				
Calf (<6 months)	18	11	61.1	0.62
Stud	15	9	60	
Dry females	134	62	46.3	
Females with 1 delivery	89	45	50.6	
Females with >1 deliveries	83	38	45.8	
Water sharing				
Yes	132	57	43.2	0.1
No	207	108	52.2	
Interaction with cattle				
Yes	142	65	45.8	0.36
No	197	100	50.8	
Abortion				
Yes	9	2	22.2	0.17
No	298	144	48.3	

Environmental and buffaloes characteristics with a *P* value equal to or less than 0.10 in the bivariate analysis included region (*P* = 0.02), municipality (*P* = 0.002), altitude (*P* = 0.002), a mean annual temperature of 27°C (*P* = 0.02), mean annual rainfall (*P* = 0.002), and water sharing (*P* = 0.10). Multivariate analysis of such characteristics showed that *T. gondii* seropositivity in buffaloes was associated only with a mean annual rainfall between 1266–1650 mm (OR = 1.84; 95% CI: 1.15-2.94; *P* = 0.01). The result of the Hosmer-Lemeshow test was 2 (*P* = 0.24) which indicates an acceptable fit of our regression model.

## Discussion

Our results indicate that water buffaloes in Veracruz, Mexico have a relatively high seroprevalence of *T. gondii* infection. The worldwide prevalence of *T. gondii* infection in water buffaloes until 2010 was reviewed previously [1,3], and seroprevalences varied from 0% to 100% by using MAT. Since then, a few additional papers were published on this subject. Antibodies to *T. gondii* were found in 1715 (35.5%) of 4796 water buffaloes from northern Brazil. They used an in-house ELISA and the cut-off titer for IFAT was 1:40 [7]. In a report from southern Brazil, antibodies were found in 46 (27.2%) of 169 water buffaloes by IFAT; sera were run at 1:64 and 1:256 dilutions and the seropositivity results were based on a titer of 1:64 [8]. In a study in Argentina, researchers found *T. gondii* antibodies in 127 (25.4%) of 500 buffaloes by IFAT using a 1:100 cut off titer [9]. Using the same MAT that we used, *T. gondii* antibodies were found in 53 (14.3%) of 300 water buffaloes in Iran; most of the buffaloes had low titers (1:25 in 21, 1:50 in 12, 1:100 in 6, 1:200 in 2 and 1:400 in 2) [10].

In most parts of the world water buffaloes are domesticated and probably acquire *T. gondii* by ingesting food or water contaminated with oocysts shed by infected domestic cats. Water buffaloes in the present report ranged freely and had contact with domestic and wild cats. Further research is needed to understand the dynamics of feline-buffalo transmission under free-ranging conditions in Veracruz, Mexico.

In the present study water buffaloes living in municipalities with a mean annual rainfall of 1266–1650 mm had a higher seroprevalence than those living in municipalities with higher mean annual rainfall (2316–2900 mm). It is not clear why a lower mean annual rainfall is associated with higher seroprevalence of *T. gondii* infection. It is possible that a higher mean annual rainfall might be accompanied with higher erosion of contaminated soil. In addition, copious rainfalls may dilute oocysts in contaminated water that might in turn lower the risk for *T. gondii* infection. Although we are unable to explain why other environmental factors (climate, altitude, and mean annual temperature) were associated with *T. gondii* infection, it has been postulated that shifts in environmental conditions can increase the prevalence of toxoplasmosis in susceptible hosts [11]. Environmental factors influencing the degree of oocysts exposure may also cause the difference in seropositivity among ranches as noted in water buffaloes from Trinidad [12]. There are no data regarding seroprevalence of *T. gondii* infection in cats among municipalities in Veracruz State. It would be of interest to test domestic and wild cats in the region for *T. gondii* infection.

Nothing is known of the validity of different serological tests used for the diagnosis of toxoplasmosis in buffaloes. We are not aware of any serious attempts at isolation of viable *T. gondii* from tissues of naturally exposed buffaloes. Buffaloes, like cattle, are considered resistant to clinical toxoplasmosis and we are not aware of any report of clinical toxoplasmosis in buffaloes [1]. Based on studies with naturally and experimentally infected cattle, the MAT gave the best results for determining antibodies to *T. gondii* in cattle [13,14], and titers < 1:100 were considered nonspecific. A comparison of serological and bioassay data on naturally infected buffaloes is required to determine if the same criteria apply for MAT diagnosis of *T. gondii* infection in water buffaloes. The reported high excretion of viable *T. gondii* in milk of naturally infected buffaloes from Iran by Dehkordi et al. [4] needs confirmation. Further research should be conducted to determine the role of consumption of raw or undercooked meat from buffaloes in the transmission of *T. gondii* infection to humans.

## Conclusion

Results of the present study indicate that buffaloes in Veracruz State have a high *T. gondii* exposure. Further research is needed to determine the risk of ingestion of raw or undercook buffalo meat for *T. gondii* infection in humans.
